# Nanopore Analysis of Wild-Type and Mutant Prion Protein (PrP^C^): Single Molecule Discrimination and PrP^C^ Kinetics

**DOI:** 10.1371/journal.pone.0054982

**Published:** 2013-02-05

**Authors:** Nahid N. Jetha, Valentyna Semenchenko, David S. Wishart, Neil R. Cashman, Andre Marziali

**Affiliations:** 1 Department of Physics and Astronomy, University of British Columbia, Vancouver, British Columbia, Canada; 2 National Institute for Nanotechnology, Edmonton, Alberta, Canada; 3 Departments of Computing Science and Biological Sciences, University of Alberta, Edmonton, Alberta, Canada; 4 Brain Research Centre, University of British Columbia, Vancouver, British Columbia, Canada; Consejo Superior de Investigaciones Cientificas, Spain

## Abstract

Prion diseases are fatal neurodegenerative diseases associated with the conversion of cellular prion protein (PrP^C^) in the central nervous system into the infectious isoform (PrP^Sc^). The mechanics of conversion are almost entirely unknown, with understanding stymied by the lack of an atomic-level structure for PrP^Sc^. A number of pathogenic PrP^C^ mutants exist that are characterized by an increased propensity for conversion into PrP^Sc^ and that differ from wild-type by only a single amino-acid point mutation in their primary structure. These mutations are known to perturb the stability and conformational dynamics of the protein. Understanding of how this occurs may provide insight into the mechanism of PrP^C^ conversion. In this work we sought to explore wild-type and pathogenic mutant prion protein structure and dynamics by analysis of the current fluctuations through an organic α-hemolysin nanometer-scale pore (nanopore) in which a single prion protein has been captured electrophoretically. In doing this, we find that wild-type and D178N mutant PrP^C^, (a PrP^C^ mutant associated with both Fatal Familial Insomnia and Creutzfeldt-Jakob disease), exhibit easily distinguishable current signatures and kinetics inside the pore and we further demonstrate, with the use of Hidden Markov Model signal processing, accurate discrimination between these two proteins at the single molecule level based on the kinetics of a single PrP^C^ capture event. Moreover, we present a four-state model to describe wild-type PrP^C^ kinetics in the pore as a first step in our investigation on characterizing the differences in kinetics and conformational dynamics between wild-type and D178N mutant PrP^C^. These results demonstrate the potential of nanopore analysis for highly sensitive, real-time protein and small molecule detection based on single molecule kinetics inside a nanopore, and show the utility of this technique as an assay to probe differences in stability between wild-type and mutant prion proteins at the single molecule level.

## Introduction

Prion diseases are a class of fatal neurodegenerative diseases affecting both humans and animals that are associated with the accumulation of PrP^Sc^ in the central nervous system [1], [2], [3], [4], a pathological isoform of normal cellular prion protein (PrP^C^). The widely accepted protein-only hypothesis of prion disease pathogenesis implicates PrP^Sc^ as the principal and possibly sole infectious agent, capable of self-replication by post-translational interaction with PrP^C^ stimulating its conversion into PrP^Sc^ (a process known as template-directed conversion) [1], [2], [3]. The mechanics of template-directed conversion, however, are almost entirely unknown, stymied by the lack of an atomic-level structure for PrP^Sc^, primarily due to its insolubility and tendency to aggregate [5], [6]. A number of pathogenic PrP^C^ mutants exist that are characterized by an increased propensity for conversion into PrP^Sc^ and that differ from wild-type by only a single amino-acid point mutation in their primary structure. These mutations are known to perturb the stability properties and conformational dynamics of the protein [7], [8], [9]. Understanding of how this occurs may provide insight into the mechanism of PrP^C^ conversion in disease. In this work we sought to explore prion protein structure and dynamics, for both wild-type and pathogenic mutant PrP^C^ by analysis of the current fluctuations through a nanometer-scale pore in which a single prion protein has been captured. This method of biomolecule analysis, known as single-molecule nanopore analysis, has emerged as a powerful tool for investigating and characterizing the structure and dynamics of individual biomolecules [10], [11], [12], [13]. The technique involves electrophoretically driving an individual biomolecule (immersed in an electrolyte) into a nanometer-scale pore (nanopore) formed in an insulating membrane (e.g. an organic nanopore formed in a lipid bilayer). Direct monitoring of the ionic current through the pore enables detection of individual biomolecule capture events which are characterized by a substantial reduction in the nanopore current relative to an open-pore. Once captured, the ionic current through the pore serves as an extremely sensitive metric and rich source of information on the conformational dynamics and structural properties of the captured molecule [11], [12], [13]. To date, single-molecule nanopore analysis has been predominantly applied towards characterizing the structure and properties of individual DNA molecules. This has been primarily due to the enormous potential of nanopore analysis to form the basis of a low-cost, high-throughput DNA sequencing technology [14], [15]. Analysis of proteins and polypeptides by comparison has only recently begun and has shown much promise as a means by which to probe the unfolding kinetics of proteins [16], [17], characterize protein-pore interactions [18], [19], [20] and to study the transport properties of proteins through pores [18], [21], [22]. A major challenge, however, with nanopore protein analysis (in comparison to nucleic acid analysis for example) is that in contrast to heavily charged biopolymers such as DNA, the charge distribution of proteins and polypeptides can be highly irregular, positive, negative or neutral, significantly affecting the ability to capture proteins in the pore. Here we present results demonstrating capture of individual wild-type and D178N mutant PrP^C^ molecules into an organic α-hemolysin nanopore (mutant D178N is a pathogenic PrP^C^ mutant associated with both Fatal Familial Insomnia and Creutzfeldt-Jakob disease [23], [24]). We show that these two proteins, which differ from each other by only a single amino-acid point mutation in their primary structure, exhibit easily distinguishable current signatures and kinetics inside the pore and we further demonstrate, with the use of Hidden Markov Model signal processing, accurate detection and discrimination between these two proteins at the single molecule level based on the kinetics of a single PrP^C^ capture event. In addition, we present a four-state model to describe wild-type PrP^C^ kinetics in the pore which represents a first step in our investigation into characterizing the differences in kinetics and conformational dynamics between wild-type and D178N mutant PrP^C^.

## Materials and Methods

### PrP^C^ Constructs

PrP^C^ (both wild-type and mutant) was expressed and purified by the PrioNet Prion Protein & Plasmid Production Platform Facility (refer to File S1 for details on expression and purification protocol).Truncated Syrian Hamster PrP^C^ (residues 120–232– designated ShPrP(120–232)) was used in order to investigate the structure and dynamics of the PrP^C^ structural core. To facilitate capture of PrP^C^ inside the nanopore, the N-terminus of ShPrP(120–232) was adapted with four positively charged amino-acid residues (KKRR) (designated KKRR-ShPrP(120–232)). We expect these additional residues to have a minimal effect on the overall structure and stability of PrP^C^ based on previous studies whereby truncated PrP^C^ (of various lengths) was adapted with a 22 residue N-terminal fusion tag, which was found to have no influence on PrP^C^ structural stability [25], [26], [27]. Three PrP^C^ constructs were investigated in this study, namely: ShPrP(120–232), KKRR-ShPrP(120–232) and KKRR-ShPrP(120–232)-D178N (i.e. mutant PrP^C^).

### Nanopore Experiments

**Figure 1:**
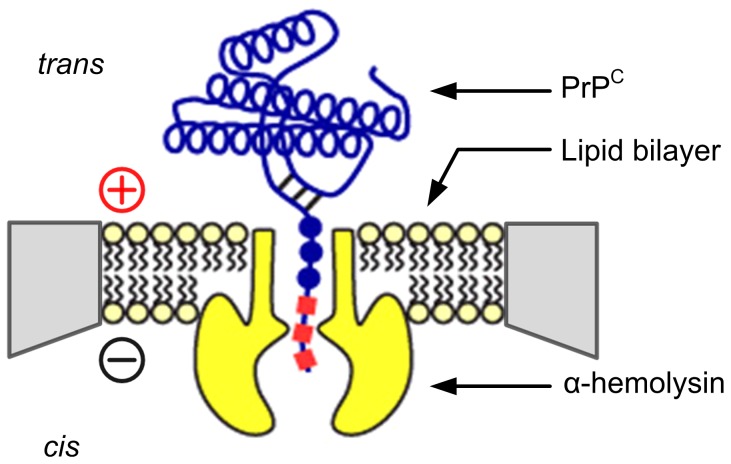
Cartoon illustrating the capture of an individual PrP^C^ molecule into an α-hemolysin nanopore. PrP^C^ is electrophoretically driven into the α-HL nanopore (voltage polarity given by the plus and minus signs) via its positively charged N-terminus. The *trans* chamber contains 0.3 M KCl, 45 mM NaPO_4_, 10 mM HEPES, pH 8.0 solution at a PrP^C^ concentration of 13.4 µM. The *cis* chamber contains 3 M KCl, 10 mM HEPES, pH 8.0 solution. The salt-concentration gradient across the pore generates an osmotic flow from *trans*-to-*cis* and enhances the electric field around the entrance of the *trans*-side of the pore [30], [31] thereby substantially increasing the nanopore-capture rate of PrP^C^ in solution relative to symmetric salt conditions [30], [31]. Experiments were conducted (and maintained) at a temperature of 20°C ±0.1°C.

α-hemolysin (α-HL) nanopores were formed using a method adapted from that of Akeson et al. [28]. Briefly, a black lipid membrane of 1,2-diphytanoyl-sn-glycero-3-phosphocholine (Avanti Polar Lipids Inc., Alabaster, AL) and hexadecane (Sigma-Aldrich, St. Louis, MO) is formed across a 25 µm PTFE aperture connecting two baths filled with electrolyte (Figure 1. Details on lipid bilayer formation are described elsewhere [29]). Owing to the low charge density of KKRR-ShPrP(120–232) (relative to heavily charged biopolymers such as DNA and RNA), and thus decreased ability to capture PrP^C^ in the pore, experiments were conducted under asymmetric salt conditions which, through a combination of electric field enhancement around the entrance of the pore and osmotic flow [30], [31], substantially increases the nanopore-capture rate of small molecules in solution (relative to symmetric salt conditions) [30], [31]. PrP^C^ was preloaded on the *trans* side of the pore in 0.3 M KCl, 45 mM NaPO_4_, 10 mM HEPES, pH 8.0 solution (final PrP^C^ concentration was ∼13.4 µM). We do not expect oligomerization, aggregation or precipitation of PrP^C^ under these solution conditions based on mass spec results of KKRR-ShPrP(120–232) in solution and on previous studies with various full-length and truncated forms of PrP^C^ in high salt [32], [33]. Our mass spec data confirms the presence of monomeric PrP^C^ in solution and the absence of dimers (data not shown), suggesting the absence of higher-order oligomers as well. Evidence in the literature indicates that the aggregation propensity of PrP^C^ in the presence of high salt is due to interactions of anions in solution with glycine groups in the glycine-rich unstructured PrP^C^ N-terminus (i.e. residues 23–119), and is therefore a property of full-length PrP^C^ (i.e. PrP^C^(23–232)) [32], [33]. Moreover, studies of truncated PrP^C^ (i.e. PrP^C^(120–232)) in high salt buffer (0.5 M NaCl) find no change in secondary structure content compared to salt-free buffer and do not report of aggregation or precipitation of PrP^C^ in solution [32]. In contrast to the *trans* side of the pore the *cis* side contained 3 M KCl, 10 mM HEPES, pH 8.0 solution. All experiments were conducted (and maintained) at a temperature of 20°C ±0.1°C. An Axon Axopatch 200 B patch clamp amplifier is used to measure the ionic current. Data is low-pass filtered at 10 kHz by a 4-pole Bessel filter and sampled at 100 kHz. Experiments were conducted with a single α-HL pore incorporated in the lipid bilayer. Formation of an α-HL pore was done under symmetric salt conditions (i.e. 0.3 M KCl on both the *cis* and *trans* sides of the pore) by injection of free subunits into solution (on the *cis* side of the pore) which subsequently self-assemble into heptameric, membrane-spanning pores or by injection of preformed heptameric α-HL into solution which spontaneously forms into a transmembrane pore in a lipid bilayer [34]. Confirmation of a single pore incorporation is achieved by applying a +100 mV electric potential across the membrane (*trans* side positive) and observing a specific step-wise increase in the current (∼+28 pA at +100 mV and ∼−20 pA at −100 mV). Once this is observed, the RMS current noise on the pore (5 kHz bandwidth using a Butterworth filter – independent of the 10 kHz low-pass Bessel filter) is then probed at +100 mV and +200 mV to confirm that a heptameric pore has incorporated into the bilayer (as opposed to an anomalous structure, e.g. a hexameric pore). At +100 mV the upper limit for the 5 kHz noise (indicative of a heptameric pore), for our experimental setup, is ∼0.80 pA RMS, and at +200 mV the upper limit is ∼1.20 pA RMS. If both the current and noise are within specification, the *cis* side of the pore is then perfused with 3 M KCl, 10 mM HEPES, pH 8.0 solution several times to ensure that any free, unbound α-HL has been removed from solution and to ensure that the salt concentration on the *cis*-side of the pore has been increased to 3 M KCl. Custom-built data acquisition software (described elsewhere [35]) is used to apply large positive voltages (e.g. +160 mV or greater) across the pore (and record the corresponding current and voltage) thereby electrophoretically driving individual PrP^C^ molecules in solution into the pore. Several thousand individual PrP^C^ capture events were recorded at each voltage in the range of +160 mV to +240 mV for each PrP^C^ construct.

### PrP^C^ Capture Rate & KKRR-ShPrP (120–232) Event Lifetime

**Figure 2:**
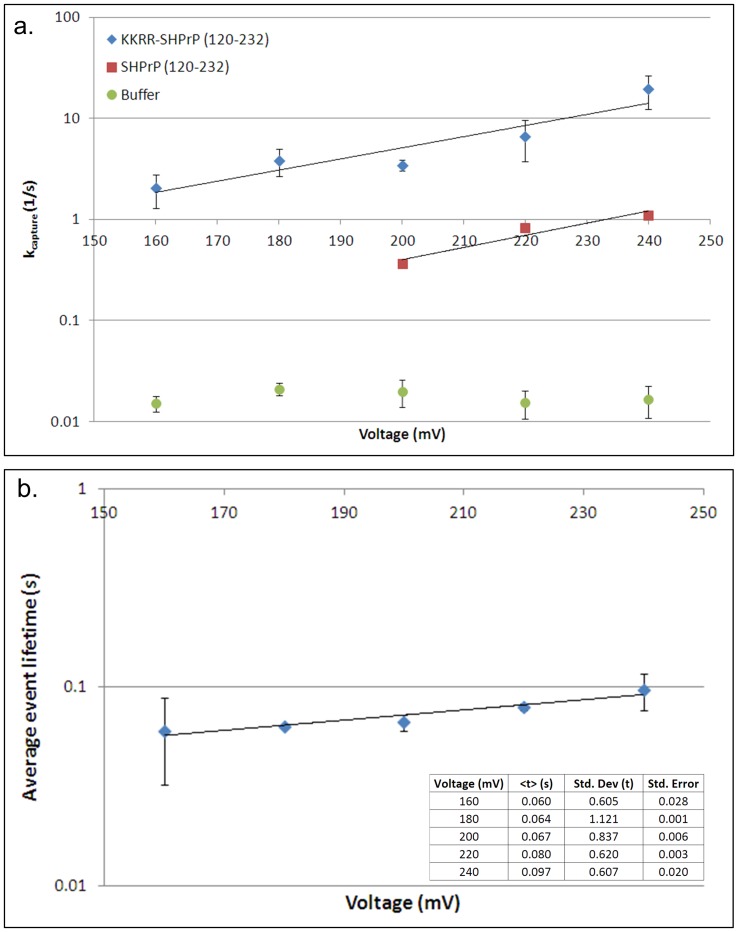
Nanopore capture rate and KKRR-ShPrP(120–232) average event lifetime as a function of voltage (3 **M KCl** ***cis*** **, 0.3** **M KCl** ***trans*** **).** (**a**) Shown is the capture rate as a function of voltage for KKRR-ShPrP(120–232) (blue), ShPrP(120–232) (red) and the buffer-only control (green). In the case of the buffer-only control the capture rate represents the rate of pore gating as a function of voltage. Both KKRR-ShPrP(120–232) and ShPrP(120–232) exhibit capture kinetics that are exponentially dependent on voltage consistent with the applied voltage acting on the positively charged residues at the N-terminus (five in the case of KKRR-ShPrP(120–232) and one in the case of ShPrP(120–232)) to decrease the energy barrier height for entry into the pore, thereby exponentially increasing the capture rate. In addition, the capture rate for KKRR-ShPrP(120–232) is between one and one-and-a-half orders of magnitude higher than ShPrP(120–232) indicating that the large majority of captures of KKRR-ShPrP(120–232) involve threading of the N-terminus through the pore. Error bars represent the standard error on the mean, and were determined via bootstrapping in the case of ShPrP(120–232) and the buffer-only control, whereas in the case of KKRR-ShPrP(120–232) error bars were determined based on two separate datasets. (**b**) The average event lifetime for KKRR-ShPrP(120–232) increases exponentially with voltage consistent with PrP^C^ escape from the pore (as opposed to translocation) over an electrostatic energy barrier (governed by the applied voltage). The standard deviation of the event lifetime distribution indicates the presence of both short and very long time events (i.e. >1 s). Error bars represent standard error on the mean and were determined based on two separate datasets.

To confirm that KKRR-ShPrP(120–232) enters the pore N-terminal first, we characterized and compared the nanopore capture rate as a function of voltage for KKRR-ShPrP(120–232), ShPrP(120–232), and a buffer only control (indicative of the pore gating rate under the given buffer conditions – i.e. asymmetric salt concentration, Figure 2A). The capture rate for KKRR-ShPrP(120–232) and ShPrP(120–232) is exponentially dependent on voltage, consistent with the capture process being dominated by an energy barrier whereby, according to classical Kramer’s theory, the applied voltage acts on the N-terminal positive charges (five in the case of KKRR-ShPrP(120–232) and one in the case of ShPrP(120–232)), decreasing the energy barrier height for entry into the pore, thereby exponentially increasing the rate of PrP^C^ capture. Moreover, the nanopore-capture rate of KKRR-ShPrP(120–232) is between one and one-and-a-half orders of magnitude higher (depending on voltage) than the capture rate of ShPrP(120–232), reflecting the greater charge density at the N-terminus in the case of KKRR-ShPrP(120–232). These results indicate that the large majority of captures of KKRR-ShPrP(120–232) involve threading of the N-terminus through the pore.

The average event lifetime for KKRR-ShPrP(120–232) as a function of voltage is shown in Figure 2B. The event lifetime increases exponentially with voltage, consistent with PrP^C^ escape from the pore (as opposed to translocation) being the dominant mode of termination of an event, and requiring crossing an electrostatic energy barrier (governed by the applied voltage). The standard deviation of the event lifetime distribution shows the lifetime spanning several orders of magnitude indicating the presence of both short and very long time events (i.e. >1 s) (refer to File S1 regarding details on how the capture rate and average event lifetime are determined).

## Results and Discussion

**Figure 3:**
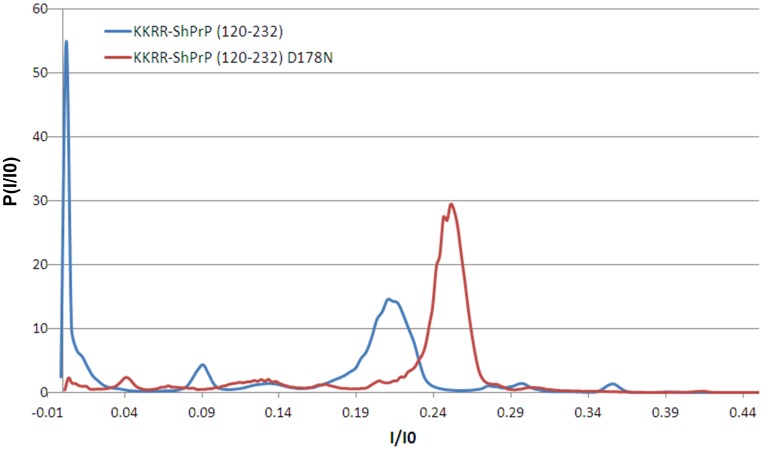
All-point current histogram for KKRR-ShPrP(120–232) and KKRR-ShPrP(120–232)-D178N. Ionic current histogram of all PrP^C^ capture events from all voltages, with ionic current normalized by the open-pore current (I0) at a given voltage, for KKRR-ShPrP(120–232) (blue) and KKRR-ShPrP(120–232)-D178N (red). Ionic current is median filtered to 2.99 ms per data-point. The histograms exhibit multiple peaks with varying degrees of overlap indicative of complex PrP^C^ kinetics in the pore. Moreover, the histograms exhibit clear distinguishable features (e.g. the near absence of the peak at I/I0 ∼0 pA with respect to KKRR-ShPrP(120–232)-D178N).

Figure 3 shows the ionic current histograms for all PrP^C^ capture events from all voltages, with ionic current normalized by the open-pore current at a given voltage, for KKRR-ShPrP(120–232) and KKRR-ShPrP(120–232)-D178N. Both histograms display multiple peaks, with varying degrees of overlap, indicative of complex PrP^C^ kinetics inside the pore. Moreover, the histograms exhibit clear distinguishable features (e.g. the near absence of the peak at I/I0 ∼0 pA with respect to mutant PrP^C^), indicating differences in conformational dynamics between the two proteins in the pore.

**Figure 4:**
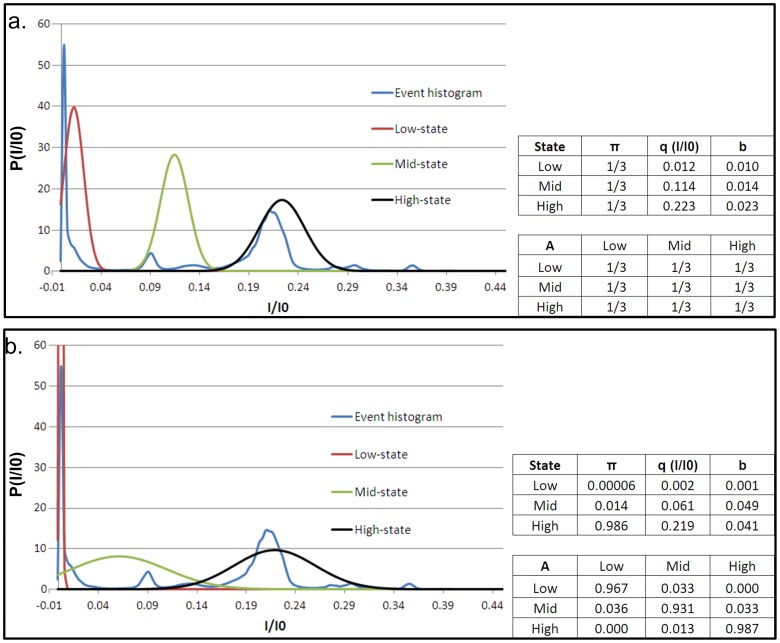
KKRR-ShPrP(120–232) event histogram and initial and optimal HMM models. (**a**) **(Left)** The KKRR-ShPrP(120–232) event histogram (blue) is divided into three regimes/states a high-state (black), mid-state (green) and low-state (red). The location of the peak and width of the distribution for each state in our initial model represents our best guess of the location and size of a given regime. **(Right)** The model parameters: *π* (i.e. the initial condition or probability that an event begins in a given state), *q* (the location of the peak of the Gaussian distribution, in terms of I/I0, for a given state), *b* (the standard deviation on the Gaussian of each state, which defines a state’s noise properties), and *A* (the state-to-state transition probability matrix). In our initial model we assume ignorance of the probabilities and therefore assume *π* to be uniformly distributed (i.e. an event is assumed equally likely to begin in any of the three states). Similarly with the transition probability matrix *A*, we assume all transitions to be equally likely (e.g. if in the low-state there is an equal probability for remaining in the low-state as there is for transitioning into the mid-state or the high-state). (**b**) **(Left)** After 40 iterations of the EM algorithm the optimal three-state model that best describes the data (i.e. the maximum likelihood model estimate) is converged upon. The low-state, far from encompassing all of the low current (as was presumed in our initial model) is very narrow and well defined, while the mid and high states both broaden out (the peak of the mid-state also shifts to a deeper conductance level relative to the initial model). **(Right)** The corresponding optimal model parameters.

**Figure 5:**
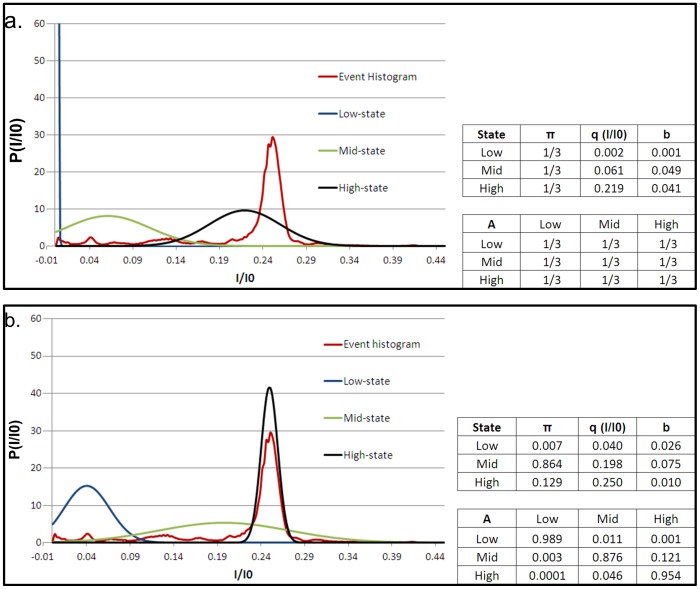
KKRR-ShPrP(120–232)-D178N event histogram and initial and optimal HMM models. (**a**) **(Left)** The KKRR-ShPrP(120–232)-D178N event histogram (red), and the corresponding high-state (black), mid-state (green) and low-state (blue) that make up the initial model for HMM analysis. The location of the peak (*q*) and width of the distribution (*b*) for each state are the same as for the optimal KKRR-ShPrP(120–232) model (Figure 4B). This choice for *q* and *b* serves to highlight how the individual states evolve and differ from that of wild-type PrP^C^. **(Right)** The corresponding initial model parameters. Similar to the initial model for KKRR-ShPrP(120–232) (Figure 4A) we assume ignorance of the probabilities and therefore assume *π* to be uniformly distributed. Likewise, with the transition probability matrix (*A*), we assume all transitions to be equally likely. (**b**) **(Left)** After 36 iterations of the EM algorithm the optimal three-state model that best describes the data (i.e. the maximum likelihood model estimate) is converged upon. The individual states (properties and kinetics) are significantly different from wild-type PrP^C^ (Figure 4B), highlighting the importance of amino-acid residue D178 to the dynamics and structural stability of PrP^C^ **(Right)** The corresponding optimal model parameters.

In order to model PrP^C^ kinetics in the pore and characterize signal statistics we developed a signal processing algorithm based on Hidden Markov Models (HMMs). HMM signal processing is a powerful technique by which to extract and characterize low-level signals buried in background noise [36], [37], [38]. The approach has been previously applied in characterizing the complex kinetics of DNA hairpins trapped in the α-HL nanopore [39], [40]. HMM analysis requires an initial model for the system defined by the following parameters: State levels in terms of I/I0 (*q*), the initial condition (*π*) (i.e. the probability that an event begins in a given state), the transition probabilities between states (*A*), and the noise properties (the standard deviation on a Gaussian) of each state (*b*). These parameters represent best guesses and can be estimated, with respect to *q* and *b*, from the event histogram. Once the initial model is developed and provided with the corresponding data, the HMM operates through an expectation maximization (EM) algorithm improving upon the model parameters with each iteration until convergence to an optimal set of parameters that best describes the data, based on model likelihood, i.e. HMM analysis converges to the maximum likelihood model estimate (we refer the reader to [36], [41] for details regarding HMM theory and its implementation). We model PrP^C^ kinetics in the pore as a three-state system. Our choice of three states to describe PrP^C^ kinetics is based on parsimony i.e. selecting a model with the fewest parameters that describes the data well. Moreover, our choice is based on wild-type PrP^C^ kinetics and in particular on a qualitative assessment of the form of the wild-type histogram. As mentioned previously, the histogram displays multiple peaks, with varying degrees of overlap. We find, however, that the peaks are concentrated into roughly three regimes. A simple description of the histogram, therefore, is one in which the current is split into a high, mid and low regime (i.e. three states - refer to Figure 4A, which shows our initial wild-type PrP^C^ HMM model and best guess at the location and size of each regime). For ease of comparison between wild-type and D178N mutant PrP^C^ we model mutant kinetics in the pore as a three-state system as well. The initial and optimal (post-HMM processed) models are shown in Figures 4 and 5 for KKRR-ShPrP(120–232) and KKRR-ShPrP(120–232)-D178N respectively.

The optimal three-state model for wild-type and mutant PrP^C^ (Figures 4B and 5B respectively) reveal significant differences in both state properties and kinetics between the two proteins, highlighting the importance of amino acid residue D178 to the dynamics and structural stability of PrP^C^. It is known that residue D178 in wild-type PrP^C^ stabilizes the protein through salt-bridge interactions with R164 (the C-terminus of β-strand 2) and by hydrogen bonding to Y128 (the N-terminal Tyr of β-strand 1) [42], [43], [44], [45]. In mutant D178N therefore, these stabilizing forces are no longer present, the loss of which appears to significantly affect the conformational dynamics of mutant PrP^C^ in the pore.

The clear distinction between these two proteins also highlights the sensitivity of nanopore analysis in detecting changes in biomolecule structure and demonstrates the potential of using this technique for detection and identification of small molecules and proteins in solution based on differences in kinetics in a nanopore. In order to explore this potential we characterized protein-calling accuracy between wild-type and D178N mutant PrP^C^ at the single event level, based on kinetic differences in the pore (i.e. given a single event we characterized the accuracy with which it can be determined which protein, either wild-type or mutant, produced the event based on kinetics). In this regard we investigated two cases in particular:

1. Where all events are analyzed, regardless of event lifetime and
2. Where only those events that have a lifetime of ≥1 s are analyzed

Case 2 allows us to characterize how protein-calling accuracy changes as we limit our study to long-time events (i.e. those events with long observation times and therefore better statistics for discriminating between the two proteins). In order to make individual calls given an event we developed a protein-calling algorithm based on HMM model likelihood. The method by which we characterize protein-calling accuracy and the results obtained are described in the following.

### Case 1

Of all wild-type and mutant PrP^C^ events we first simulate a 50∶50 mix dataset. Therefore 500 wild-type events and 500 mutant events are randomly selected and combined to form a simulated 50∶50 mix of 1000 events. The remaining events for both wild-type and mutant form the training sets for the corresponding protein by which to build optimal HMM models (refer to Figures S1 and S2 in File S1 for the determined optimal wild-type and mutant models, respectively). Protein-calling accuracy is assessed by calling the individual events from the 50∶50 mix dataset (i.e. the blind, unanalyzed events). Individual events are called as either wild-type or mutant using our HMM protein-calling algorithm. The algorithm works as follows: Given an individual event and the optimal wild-type and mutant HMM models (as determined via HMM analysis of the respective training data) the algorithm calculates the likelihood function of the event given each model (i.e. P(E|λ_wild-type_) and P(E|λ_mutant_), where *E* is the event and *λ* represents a given model). The algorithm then calls an event as either wild-type or mutant based on maximum likelihood (i.e. the protein-call is based on whichever of the two determined likelihood functions is largest). The protein-calling results are given in Table 1. The results are given in terms of the wild-type and mutant predictive value. This is defined as the likelihood that an event which is called as either wild-type or mutant is called correctly (e.g. if the algorithm calls an event as mutant there is a 0.86 likelihood that the event is a mutant event).

**Table 1:** Wild-type and mutant predictive value.

	Case 1	Case 2
# of events in 50∶50 mix	1000	176
Wild-type predictive value	0.71	0.85
Mutant predictive value	0.86	0.90

Case 1 refers to the situation whereby all events, regardless of event lifetime, are analyzed. The simulated 50∶50 mix dataset forms the individual events to be protein-called by which wild-type and mutant predictive value is determined. These events are randomly selected from the total number of wild-type and mutant events. After selection (and removal from the total number of events), the remaining events form the training sets for both wild-type and mutant. Wild-type predictive value refers to the likelihood that an event is in fact a wild-type event given that the protein-calling algorithm has called it as wild-type. Similarly the mutant predictive value refers to the likelihood that an event is in fact a mutant event given that the protein-calling algorithm has called it as mutant. Case 2 refers to the case whereby only those events that have an event lifetime of ≥1 s are analyzed (i.e. only long time events makeup the training sets for both wild-type and mutant and the generated 50∶50 mix dataset). The predictive value, not surprisingly, improves when only considering long-time events which is primarily due to the fact that long-time events can be better assessed in terms of their kinetics than short-time events (i.e. the amount of data available to characterize an event is proportional to the event lifetime) thereby improving protein-calling accuracy.

### Case 2

In characterizing protein-calling accuracy in case 2 the method is the same as described for case 1 with the difference being that in case 2 we are only interested in events that have an event lifetime of ≥1 s. In other words, all events making up the training sets (wild-type and mutant) and the generated 50∶50 mix dataset have an event lifetime of ≥1 s (refer to Figures S3 and S4 in File S1 for the determined optimal wild-type and mutant models in this case, respectively). The total number of events for the 50∶50 mix dataset is 176. The results, in terms of predictive value are given in Table 1.

These results show, not surprisingly, that predictive value (i.e. protein calling accuracy) improves when considering only long-time events. This is primarily due to the fact that long-time events can be better assessed in terms of their kinetics than short-time events (i.e. the amount of data available to characterize an event increases proportionately with the event lifetime) thereby improving protein-calling accuracy. In particular, the mutant at short times has a greater propensity for being called as wild-type than at long-times (i.e. mutant kinetics at short-times is less distinguishable from wild-type than at long-times). We note here that of all wild-type events, ∼92% of them have an event lifetime of <0.1 s. Similarly of all D178N mutant events, ∼66% have an event lifetime of <0.1 s. Therefore the majority of observed events (the large majority in the case of wild-type PrP^C^) are short-lived. Even in the case of short-lived events which are between 1 and 33 datapoints long (i.e. events are filtered to 2.99 ms per datapoint), with a large proportion of events having an event lifetime of ≤0.01 s (i.e. between 1 and 3 datapoints long − ∼30% of events in the case of wild-type PrP^C^), the results show that wild-type and mutant PrP^C^ are easily distinguished based on their kinetics in the pore. The results substantially improve, particularly in the case of wild-type predictive value, when only long-time events are considered. These results demonstrate that nanopore analysis in combination with HMM signal processing can be used to detect and discriminate between wild-type and mutant PrP^C^ at the single event level based on their kinetics in the pore. These results therefore show the potential of using this technique as an assay to probe differences in stability between wild-type and mutant prion proteins at the single molecule level, which opens up the possibility of studying small molecule-PrP^C^ interactions and the effects of these molecules on PrP^C^ stability as a possible screen for small molecules that improve the stability properties of the protein. Moreover, the ability to discriminate between two proteins that differ by only single-amino acid point mutation demonstrates the sensitivity of this approach in detecting subtle changes in biomolecule structure, and points to the possibility of developing this technique for highly sensitive, real-time detection and identification of small molecules and proteins in solution, with potential applications in disease biomarker and pathogen detection.

**Figure 6:**
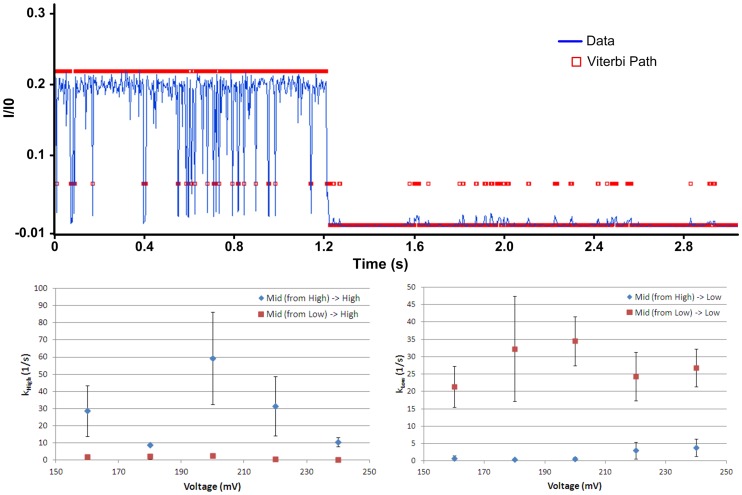
KKRR-ShPrP(120–232) mid-state statistics as a function of voltage. (Top) A sample KKRR-ShPrP(120–232) event (blue) with the most likely state sequence (i.e. Viterbi path) overlayed (red) as determined by a Viterbi analysis of the event. The sample event highlights the dependence of mid-state statistics (i.e. the transition rates out of the mid-state) on how the mid-state is entered. The event qualitatively shows that if the mid-state is entered from the high state then a transition back to the high-state is more likely than a transition into the low-state. Likewise, transitions into the mid-state from the low-state are more likely to return to the low-state as opposed to entering the high-state. **(Bottom left)** Mid-state transition rate into the high-state as a function of voltage, depending on how the mid-state is entered. If the mid-state is entered from the high-state (blue) the transition rate back into the high-state is between one and two orders of magnitude higher (depending on voltage) than the transition rate into the high-state when the mid-state is entered from the low-state (red). **(Bottom right)** Mid-state transition rate into the low-state as a function of voltage, depending on how the mid-state is entered. If the mid-state is entered from the low-state (red) the transition rate back into the low-state is between one and two orders of magnitude higher (depending on voltage) than the transition rate into the low-state when the mid-state is entered from the high-state (blue).

**Figure 7:**
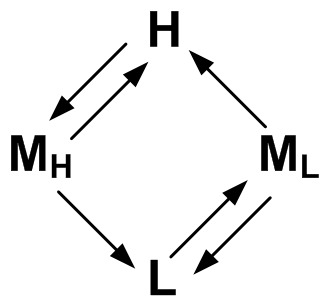
Four-state model characterizing KKRR-ShPrP(120–232) kinetics in the pore. H, M_H_, L, and M_L_ refer to the high, mid-high, low, and mid-low states respectively (N.B. PrP^C^ can escape from the pore from each state, which is not explicitly shown in the four-state model). HMM analysis of KKRR-ShPrP(120–232) in combination with the mid-state analysis (refer to text) yields the information on how the states are connected.

We return now to a more detailed analysis of PrP^C^ kinetics in the pore with the goal of characterizing the kinetic differences between wild-type and D178N mutant PrP^C^. We limit our discussion here specifically to the kinetics of wild-type PrP^C^ (see below for a discussion D178N mutant kinetics). The optimal model shown in Figure 4B is a model of the kinetics of PrP^C^ in the pore over all voltages. To characterize the voltage-dependence of PrP^C^ kinetics the voltage-specific optimal model must be determined. This is done by HMM analysis of only those events at a given voltage whereby the optimal model (Figure 4B) serves as the initial model for the voltage-specific HMM analysis, with the caveat that the state levels (*q*) and noise properties of each state (*b*) remain constant during the analysis (i.e. only *π* and *A* are updated during the voltage-specific HMM analysis). The voltage-specific HMM analysis therefore, improves the estimate of the initial condition and the transition probabilities for a given voltage. Given the voltage-specific optimal model and an individual event the most likely state sequence for the event is then determined (i.e. the event Viterbi path) via the Viterbi algorithm (refer to [41] regarding the theory and implementation of the Viterbi algorithm). State properties, such as the lifetime distribution of each state, and state-to-state transition rates are then determined by analyzing the Viterbi path for all events (refer to File S1 for details on how the state-to-state transition rates are determined). Shown in Figure 6 are the statistics of the mid-state (i.e. the transition rate from the mid-state to the high and low states as a function of voltage), highlighting the dependence of mid-state statistics on how it is entered. For example, the transition rate from the mid-state to the low-state differs by one-to-two orders of magnitude depending on if the mid-state is entered from the high-state versus if it is entered from the low-state. In general for a single state, we would expect the transition rates in Figure 6 (bottom left) and Figure 6 (bottom right) to be within an error bar of each other (assuming the rates are Gaussian distributed). Given their degree of separation, between two and four error bars depending on voltage, these results indicate that the mid-state is more accurately modeled as two separate states a mid-high and a mid-low state, to distinguish between transitions into the mid-state from the high-state (mid-high state) versus mid-state transitions from the low-state (mid-low state). Given this, together with the results from the HMM analysis of the data (i.e. the state-to-state transition probabilities), we can model KKRR-ShPrP(120–232) kinetics in the pore as a four-state system (Figure 7).

Given the detailed kinetics of wild-type PrP^C^ in the pore, the voltage-dependence of all the state-to-state transition rates can be determined, which may yield information on the different conformations of PrP^C^ in the pore. For example, transitions that exhibit an exponential dependence on voltage (i.e. Arrhenius kinetics) indicate energy barrier crossing processes and therefore yield clues on the types of conformations and conformational transitions that can makeup said processes. This together with computational modeling of PrP^C^ trapped inside the pore should reveal detailed information on the specific conformations of PrP^C^ in the pore. With respect to D178N mutant kinetics and how it compares with wild-type we find that given the substantial difference in state properties between these two constructs (i.e. Figures 4B and 5B) no simple comparisons can be made. As mentioned previously, residue D178 plays an important role in maintaining the structural stability of PrP^C^. This loss of stability, in the case of the mutant, likely enables it to adopt a variety of conformations inside the pore that are inaccessible to wild-type, which complicates the comparison between these two constructs, and hints at the need for additional states in a description of mutant kinetics in the pore. In order to make meaningful comparisons with wild-type PrP^C^, therefore, a more detailed analysis of mutant kinetics is required.

### Conclusions

We probed wild-type and D178N mutant PrP^C^ structure and dynamics by analyzing the current fluctuations through an α-HL nanopore in which a single PrP^C^ molecule has been captured electrophoretically. We have shown that these two proteins (proteins that differ by only a single amino-acid point mutation) exhibit easily distinguishable current signatures and kinetics inside the pore and have demonstrated, with the use of HMM signal processing, accurate detection and discrimination between these two proteins at the single molecule level based on the kinetics of a single PrP^C^ capture event. This method of protein analysis may be useful as an assay to probe differences in stability between wild-type and mutant prion proteins at the single molecule level, opening up the possibility to study small molecule-PrP^C^ interactions and their effects on PrP^C^ stability as a possible screen for small molecules that improve the stability properties of the protein. Moreover, our results demonstrate the sensitivity of nanopore analysis in detecting subtle changes in biomolecule structure and show its potential for highly sensitive, real-time protein and small molecule detection and identification based on single molecule kinetics inside a nanopore with potential applications in disease biomarker and pathogen detection. In addition, we developed a four-state model to characterize wild-type PrP^C^ kinetics in the pore which represents a first step in our investigation on characterizing the differences in kinetics and conformational dynamics between wild-type and D178N mutant PrP^C^, a comparison of which may ultimately yield clues into the molecular mechanism of PrP^C^ conversion in disease. These results demonstrate the ability of nanopore analysis to probe the detailed kinetics and conformational dynamics of a single biomolecule and point to the potential of using this technique in probing the molecular properties of other clinically relevant proteins (e.g. Aβ oligomers, α-synuclein, etc…).

## Supporting Information

File S1 **PrP^C^ expression and purification protocol, case 1 and case 2 optimal models, and additional details on data analysis.**
(DOCX)

## References

[1] Collinge J (2001) Prion diseases of humans and animals: Their causes and molecular basis. Annu. Rev. Neurosci. 24: 519–550. 10.1146/annurev.neuro.24.1.519 11283320

[2] Prusiner SB (1982) Novel proteinaceous infectious particles cause scrapie. Science. 216: 136–144. 10.1126/science.6801762 6801762

[3] Prusiner SB (1998) Prions. Proc. Natl. Acad. Sci. USA. 95: 13363–13383. 10.1073/pnas.95.23.13363 PMC33918 9811807

[4] Coulthart MB, Cashman NR (2001) Variant Creutzfeldt-Jakob disease: A summary of current scientific knowledge in relation to public health. Can. Med. Assoc. J. 165: 51–58. PMC81246 11468957

[5] Caughey B, Kocisko DA, Raymond GJ, Lansbury PT Jr (1995) Aggregates of scrapie-associated prion protein induce the cell-free conversion of protease-sensitive prion protein to the protease-resistant state. Chem. Biol. 2: 807–817. 10.1016/1074-5521(95)90087-x 8807814

[6] Caughey B, Raymond GJ, Kocisko DA, Lansbury PT Jr (1997) Scrapie infectivity correlates with converting activity, protease resistance, and aggregation of scrapie-associated prion protein in guanidine denaturation studies. J. Virol. 71: 4107–4110. 10.1128/jvi.71.5.4107-4110.1997 PMC191566 9094691

[7] van der Kamp MW, Daggett V (2010) Pathogenic Mutations in the Hydrophobic Core of the Human Prion Protein Can Promote Structural Instability and Misfolding. J. Mol. Biol. 404: 732–748. 10.1016/j.jmb.2010.09.060 PMC2994014 20932979

[8] Vanik DL, Surewicz WK (2002) Disease-associated F198S Mutation Increases the Propensity of the Recombinant Prion Protein for Conformational Conversion to Scrapie-like Form. J. Biol. Chem. 277: 49065–49070. 10.1074/jbc.M207511200 12372829

[9] Meli M, Gasset M, Colombo G (2011) Dynamic diagnosis of familial prion diseases supports the β2-α2 loop as a universal interference target. PLoS One. 6: e19093. 10.1371/journal.pone.0019093 PMC3084259 21552571

[10] Ma L, Cockroft SL (2010) Biological nanopores for single molecule biophysics. ChemBioChem. 11: 25–34. 10.1002/cbic.200900526 19938028

[11] DeGuzman VS, Lee CC, Deamer DW, Vercoutere WA (2006) Sequence-dependent gating of an ion channel by DNA hairpin molecules. Nucleic Acids Res. 34: 6425–6437. 10.1093/nar/gkl754 PMC1702491 17130164

[12] Manrao EA, Derrington IM, Laszlo AH, Langford KW, Hopper MK, et al (2012) Reading DNA at single-nucleotide resolution with a mutant MspA nanopore and phi29 DNA polymerase. Nat. Biotechnol. 30: 349–353. 10.1038/nbt.2171 PMC3757088 22446694

[13] Lin J, Kolomeisky A, Meller A (2010) Helix-coil kinetics of individual polyadenylic acid molecules in a protein channel. Phys. Rev. Lett. 104: 158101. 10.1103/PhysRevLett.104.158101 20482020

[14] Venkatesan BM, Bashir R (2011) Nanopore sensors for nucleic acid analysis. Nat. Nanotechnol. 6: 615–624. 10.1038/nnano.2011.129 21926981

[15] Branton D, Deamer DW, Marziali A, Bayley H, Benner SA, et al (2008) The potential and challenges of nanopore sequencing. Nat. Biotechnol. 26: 1146–1153. 10.1038/nbt.1495 PMC2683588 18846088

[16] Payet L, Martinho M, Pastoriza-Gallego M, Betton JM, Auvray L, et al (2012) Thermal unfolding of proteins probed at the single molecule level using nanopores. Anal. Chem. 84: 4071–4076. 10.1021/ac300129e 22486207

[17] Merstorf C, Cressiot B, Pastoriza-Gallego M, Oukhaled A, Betton JM, et al (2012) Wild-type, mutant protein unfolding and phase transition detected by single nanopore recording. ACS Chem. Biol. 7: 652–658. 10.1021/cb2004737 22260417

[18] Movileanu L, Schmittschmitt JP, Scholtz JM, Bayley H (2005) Interactions of peptides with a protein pore. Biophys. J. 89: 1030–1045. 10.1529/biophysj.104.057406 PMC1366589 15923222

[19] Mohammad MM, Movileanu L (2008) Excursion of a single polypeptide into a protein pore: Simple physics but complicated biology. Eur. Biophys. J. 37: 913–925. 10.1007/s00249-008-0309-9 18368402

[20] Mohammad MM, Prakash S, Matouschek A, Movileanu L (2008) Controlling a single protein in a nanopore through electrostatic traps. J. Am. Chem. Soc. 130: 4081–4088. 10.1021/ja710787a 18321107

[21] Soskine M, Biesemans A, Moeyaert B, Cheley S, Bayley H, et al (2012) An Engineered ClyA Nanopore Detects Folded Target Proteins by Selective External Association and Pore Entry. Nano Lett. 12: 4895–4900. 10.1021/nl3024438 PMC3440510 22849517

[22] Pastoriza-Gallego M, Rabah L, Gibrat G, Thiebot B, Gisou van der Gout F, et al (2011) Dynamics of unfolded protein transport through an aerolysin pore. J. Am. Chem. Soc. 133: 2923–2931. 10.1021/ja1073245 21319816

[23] Goldfarb LG, Petersen RB, Tabaton M, Brown P, LeBlanc AC, et al (1992) Fatal familial insomnia and familial Creutzfeldt Jakob disease: disease phenotype determined by a DNA polymorphism. Science 258: 806–808. 1439789 10.1126/science.1439789

[24] Monari L, Chen SG, Brown P, Parachi P, Petersen RB, et al (1994) Fatal familial insomnia and familial Creutzfeldt-Jakob disease: different prion proteins determined by a DNA polymorphism. Proc. Natl. Acad. Sci. USA. 91: 2839–2842. 10.1073/pnas.91.7.2839 PMC43466 7908444

[25] Julien O, Chatterjee S, Bjorndahl TC, Sweeting B, Acharya S, et al (2011) Relative and regional stabilities of the hamster, mouse, rabbit, and bovine prion proteins toward urea unfolding assessed by nuclear magnetic Resonance and circular dichroism spectroscopies. Biochemistry. 50: 7536–7545. 10.1021/bi200731e 21800884

[26] Bjorndahl TC, Zhou G, Liu X, Perez-Pineiro R, Semenchenko V, et al (2011) Detailed biophysical characterization of the acid-induced PrP^C^ to PrP^β^ conversion process. Biochemistry. 50: 1162–1173. 10.1021/bi101435c 21189021

[27] Perez-Pineiro R, Bjorndahl TC, Berjanskii MV, Hau D, Li L, et al (2011) The prion protein binds thiamine. FEBS J. 278: 4002–4014. 10.1111/j.1742-4658.2011.08304.x 21848803

[28] Akeson M, Branton D, Kasianowicz JJ, Brandin E, Deamer DW (1999) Microsecond timescale discrimination among polycytidylic acid, polyadenylic acid, and polyuridylic acid as homopolymers or as segments within single RNA molecules. Biophys. J. 77: 3227–3233. 10.1016/S0006-3495(99)77153-5 PMC1300593 10585944

[29] Jetha N, Wiggin M, Marziali A (2009) Forming an α-hemolysin nanopore for single molecule analysis. In: Lee JW, Foote RS, editors. Micro and Nano Technologies in Bioanalysis. Humana Press, Totowa NJ. 113–127. 10.1007/978-1-59745-483-4_9 19488697

[30] Wanunu M, Morrison W, Rabin Y, Grosberg AY, Meller A (2010) Electrostatic focusing of unlabelled DNA into nanoscale pores using a salt gradient. Nat. Nanotechnol. 5: 160–165. 10.1038/nnano.2009.379 PMC2849735 20023645

[31] Hatlo MM, Panja D, Roij RV (2011) Translocation of DNA molecules through nanopores with salt gradients: The role of osmotic flow. Phys. Rev. Lett. 107: 068101. 10.1103/PhysRevLett.107.068101 21902370

[32] Nandi PK, Leclerc E, Marc D (2002) Unusual property of prion protein unfolding in neutral salt solution. Biochemistry. 41: 11017–11024. 10.1021/bi025886t 12206674

[33] Apetri AC, Surewicz WK (2003) Atypical effect of salts on the thermodynamic stability of human prion protein, J. Biol. Chem. 278: 22187–22192. 10.1074/jbc.M302130200 12676939

[34] Movileanu L, Cheley S, Howorka S, Braha O, Bayley H (2001) Location of a constriction in the lumen of a transmembrane pore by targeted covalent attachment of polymer molecules. J. Gen. Physiol. 117: 239–251. 10.1085/jgp.117.3.239 PMC2225620 11222628

[35] Jetha N, Wiggin M, Marziali A (2009) Nanopore force spectroscopy on DNA duplexes. In: Lee JW, Foote RS, editors. Micro and Nano Technologies in Bioanalysis. Humana Press, Totowa NJ. 129–150. 10.1007/978-1-59745-483-4_10 19488698

[36] Chung SH, Moore JB, Xia L, Premkumar LS, Gage PW (1990) Characterization of single channel currents using digital signal processing techniques based on hidden markov models. Phil. Trans. R. Soc. Lond. B. 329: 265–285. 10.1098/rstb.1990.0170 1702543

[37] Churbanov A, Winters-Hilt S (2008) Clustering ionic flow blockade toggles with a mixture of HMMs. BMC Bioinf. 9: S13. 10.1186/1471-2105-9-S9-S13 PMC2537564 18793458

[38] Venkataramanan L, Sigworth FJ (2002) Applying hidden markov models to the analysis of single ion channel activity. Biophys. J. 82: 1930–1942. 10.1016/S0006-3495(02)75542-2 PMC1301989 11916851

[39] Winters-Hilt S, Vercoutere W, DeGuzman VS, Deamer D, Akeson M, et al (2003) Highly accurate classification of watson-crick basepairs on termini of single DNA molecules. Biophys. J. 84: 967–976. 10.1016/S0006-3495(03)74913-3 PMC1302674 12547778

[40] Vercoutere WA, Winters-Hilt S, DeGuzman VS, Deamer D, Ridino SE, et al (2003) Discrimination among individual Watson-Crick base pairs at the termini of single DNA hairpin molecules. Nucleic Acids Res. 31: 1311–1318. 10.1093/nar/gkg218 PMC150236 12582251

[41] Rabiner LR (1989) A tutorial on hidden markov models and selected applications in speech recognition. Proc. IEEE. 77: 257–286.

[42] Alonso DO, DeArmond SJ, Cohen FE, Daggett V (2001) Mapping the early steps in the pH-induced conformational conversion of the prion protein. Proc. Natl. Acad. Sci. USA. 98: 2985–2989. 10.1073/pnas.061555898 PMC30593 11248018

[43] Riek R, Wider G, Billeter M, Hornemann S, Glockshuber R, et al (1998) Prion protein NMR structure and familial human spongiform encephalopathies. Proc. Natl. Acad. Sci. USA. 95: 11667–11672. 10.1073/pnas.95.20.11667 PMC21698 9751723

[44] Zuegg J, Gready JE (1999) Molecular dynamics simulations of human prion protein: Importance of correct treatment of electrostatic interactions. Biochemistry. 38: 13862–13876. 10.1021/bi991469d 10529232

[45] Barducci A, Chelli R, Procacci P, Schettino V (2005) Misfolding pathways of the prion protein probed by molecular dynamics simulations. Biophys. J. 88: 1334–1343. 10.1529/biophysj.104.049882 PMC1305135 15556981

