# Supplementary material for: Nanopore Analysis of Wild-Type and Mutant Prion Protein (PrPC): Single Molecule Discrimination and PrPC Kinetics
Source: PLoS One. 2013 Feb 5;8(2):e54982. doi: 10.1371/journal.pone.0054982 (PMC3564863; doi:10.1371/journal.pone.0054982)
Supplement: File S1 — PrPC expression and purification protocol, case 1 and case 2 optimal models, and additional details on data analysis. (DOCX) [file pone.0054982.s001.docx]

**Supplementary Information**

**PrP^C^ expression and purification**

The gene for ShPrP(120-232) fragment was purchased from DNA2.0. All reagents were purchased from Sigma and Fisher except for the nickel-NTA resin which was purchased from Qiagen. Mutagenesis was performed using standard protocol from QuikChange Site-Directed Mutagenesis Kit (Stratagene).

All genes (ShPrP(120-232), KKRR-ShPrP(120-232), and KKRR-ShPrP(120-232)-D178N) were inserted into pET15b(+) vector between *XhoI* and *EcoRI* restriction sites. Expression prion constructs were verified by DNA sequencing. Recombinant proteins were expressed in *Eschericia coli BL21(DE3)* host cells in LB medium supplemented with 100 μg/ml ampicillin. The cultures were grown at 37^o^C until an OD_600_ = 0.8 was reached and then induced with 1.0 mM IPTG for 18 hr at 37^o^C. After post-induction incubation the cells were harvested by centrifugation for 20 min at 9060 x g (4^o^C). All prion proteins were isolated from the inclusion bodies and purified via refolding on the Ni-NTA column. The cell pellets from 1L of the LB medium with expressed prion protein was re-suspended in 25ml of the lysis buffer (50mM Tris, 100mM NaCl, 5mM EDTA, 0.1% NaN**_3,_** pH 8.0) and lysed by sonication (using Fisher Scientific Ultrasonic Dismembrator Model 500 with microtip probe for 6 X15sec pulses at 40% power) on ice with addition of PMSF and Triton X-100 to final concentrations of 0.1mM and 0.5% respectively. The mixture was incubated on ice for 15min followed by centrifugation at 17400 x g for 20 min (4^o^C). The supernatant was removed and inclusion bodies were cleaned by two washing steps with lysis buffer containing 0.5% Triton X-100. Triton X-100 was removed by washing the pellet twice with lysis buffer only. Inclusion bodies were centrifuged at 17400 x g for 20 min (4^o^C) after every wash and supernatant was removed. Clean protein pellet was resuspended in 30ml of the solubilization buffer (10mM Tris, 100mM sodium phosphate, 10mM reduced glutathione, 8M Urea, pH 8.0) and left for overnight incubation at room temperature with next centrifugation at 48400 x g for 1hr at 25^o^C.

The Ni-NTA resin (40ml bed volume) was pre-equilibrated with solubilization buffer and mixed with clarified supernatant. The mixture was incubated (with slow rotation) at room temperature for 10 min and packed into the column. Unbound proteins were removed by column wash with 5 bed volumes of the solubilization buffer. Prion protein was refolded on a column by decreasing Urea concentration from 8M to 0M. Linear gradient of solubilization buffer and refolding buffer (10mM Tris, 100mM sodium phosphate, pH 8.0) was applied to the column. Non-specific bound proteins were washed out of the column with 5 bed volumes of the washing buffer (10mM Tris, 100mM sodium phosphate, 50mM Imidazole, pH 8.0). Prion protein was eluted from the column with elution buffer (10mM Tris, 100mM sodium phosphate, 500mM Imidazole, pH 5.8). Fractions containing protein were identified by SDS-PAGE gel and mixed. His-tag was removed from the prion protein using Enterokinase Cleavage Capture Kit (Novagen). Purified prion proteins were then concentrated using an Amicon Ultra-15 centrifugal device.

Protein concentration was determined using an extinction coefficient at 280nm (ShPrP(120-232) extinction coefficient is 20525M^-1^cm^-1^, KKRR-ShPrP(120-232) extinction coefficient is 20525M^-1^cm^-1^, and KKRR-ShPrP(120-232)-D178N extinction coefficient is 20525M^-1^cm^-1^).Extinction coefficients were calculated by Protparam program based on every specific recombinant prion amino acid sequence.

**PrP^C^ capture rate and average event lifetime analysis**

The nanopore capture rate (or the pore gating rate in the case of the buffer-only control) for KKRR-ShPrP(120-232) and ShPrP(120-232), at a given voltage, is determined by first calculating the survival probability, *P_s_(t)*, of the open-channel state as a function of time. This represents the probability that the pore is open (i.e. unoccupied) at time *t* since the end of the last capture event. Capture of a molecule into the pore is defined, in terms of pore-current, when I/I0 crosses a threshold of 0.84, where *I* is the current through the pore and *I0* is the open-pore current. *P_s_(t)* is given by:

$$P_{s}(t)=1-\frac{i}{N}$$

where the open-pore event times are sorted in increasing order. *i* is the index of the *i^th^* event (where the first event, i.e. the shortest event, has an index of 0) and *N* is the total number of events. The survival probability, in this case, exhibits exponential decay, and therefore can be least-squares fitted to an exponential function in order to extract the capture rate at a given voltage.

To determine the average event lifetime as a function of voltage for KKRR-ShPrP(120-232), the log-event times (at a given voltage), *ln(t)*, are histogrammed and normalized thereby expressing the histogram as a probability distribution (*p[ln(t)] -* log-event times are used because event times span several orders of magnitude). The average event lifetime is given as:

$\left\langle t\right\rangle=\frac{\int e^{\ln\left(t\right)}p\left[\ln\left(t\right)\right]d\left[\ln\left(t\right)\right]}{1-\frac{N}{N_{tot}}}$, (S1)

Where *N* is the number of events that do not end in escape from the pore (e.g. events that are terminated by reversing the voltage and forcing PrP^C^ out of the pore. These events are not timed and therefore do not contribute to *p[ln(t)]*) and *N_tot_* is the total number of events.

**Protein calling case 1: Optimal KKRR-ShPrP(120-232) and KKRR-ShPrP(120-232)-D178N models**

**KKRR-ShPrP(120-232)
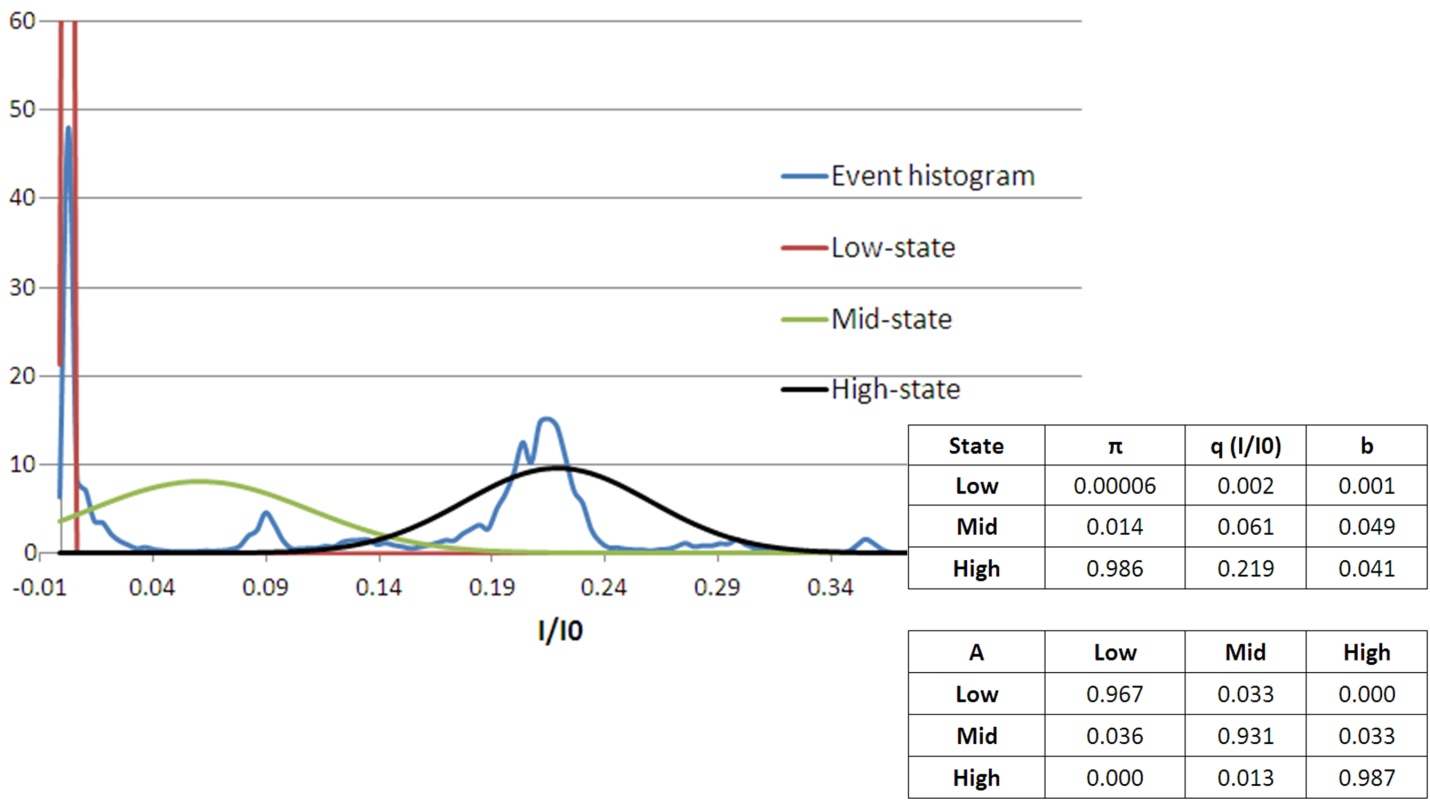
**

**Figure S1 – KKRR-ShPrP (120-232) event histogram and optimal HMM model (case 1). (Top left)** KKRR-ShPrP(120-232) event histogram of the training data (blue), with the high (black Gaussian), mid (green Gaussian), and low states (red Gaussian), as defined by the optimal HMM model to the training data, overlayed. **(Bottom right)** The optimal three-state HMM model to the training set. Initial condition (*π*), I/I0 state levels (*q*), noise properties of each state (*b* – i.e. the standard deviation on the Gaussian for each state), and the state-to-state transition probability matrix (*A*).

**KKRR-ShPrP(120-232)-D178N**

**
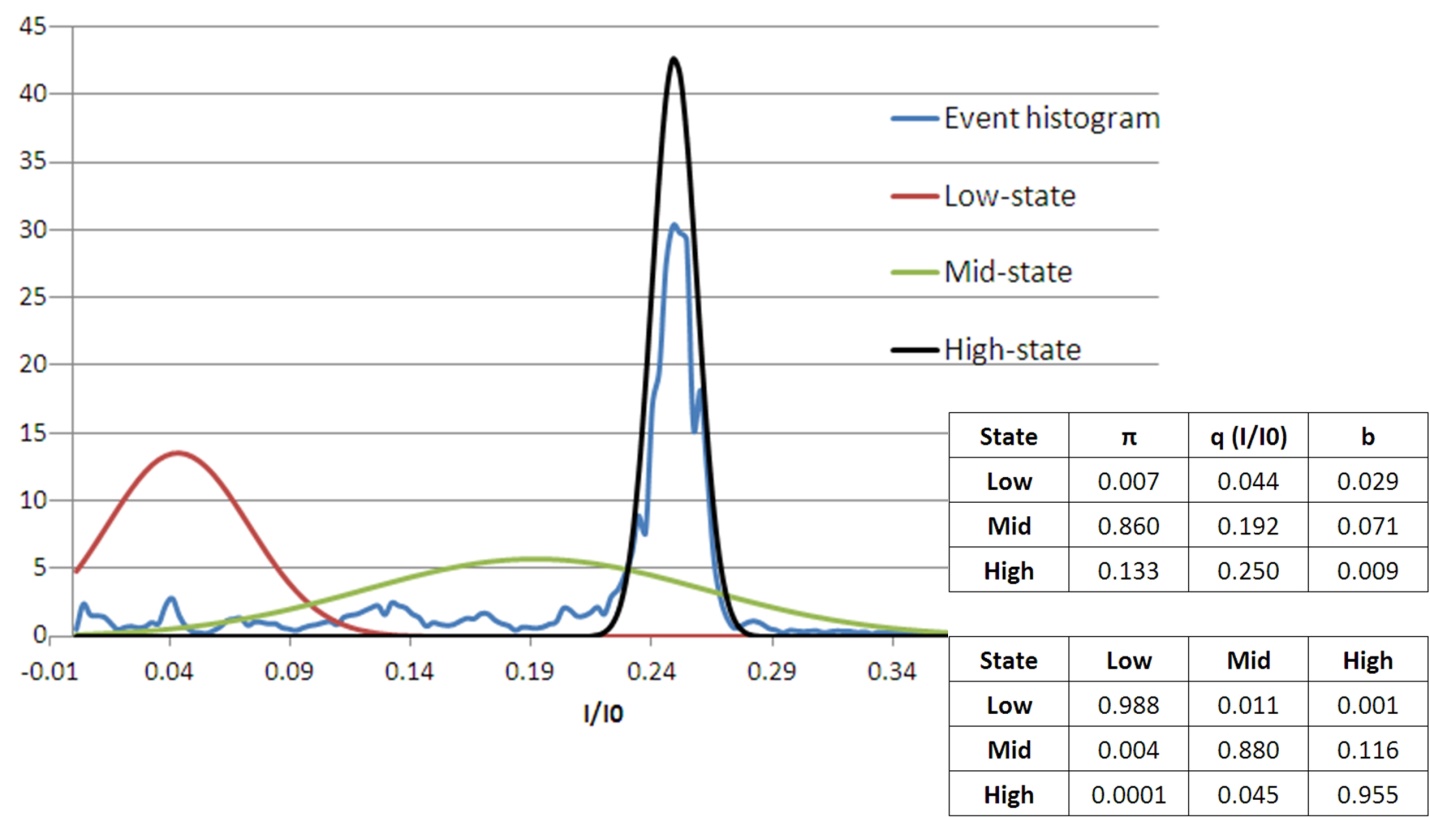
**

**Figure S2 – KKRR-ShPrP (120-232)-D178N event histogram and optimal HMM model (case 1). (Top left)** KKRR-ShPrP(120-232)-D178N event histogram of the training data (blue), with the high (black Gaussian), mid (green Gaussian), and low states (red Gaussian), as defined by the optimal HMM model to the training data, overlayed. **(Bottom right)** The optimal three-state HMM model to the training set. Initial condition (*π*), I/I0 state levels (*q*), noise properties of each state (*b* – i.e. the standard deviation on the Gaussian for each state), and the state-to-state transition probability matrix (*A*).

**Protein calling case 2: Optimal KKRR-ShPrP(120-232) and KKRR-ShPrP(120-232)-D178N models**

**KKRR-ShPrP(120-232)**


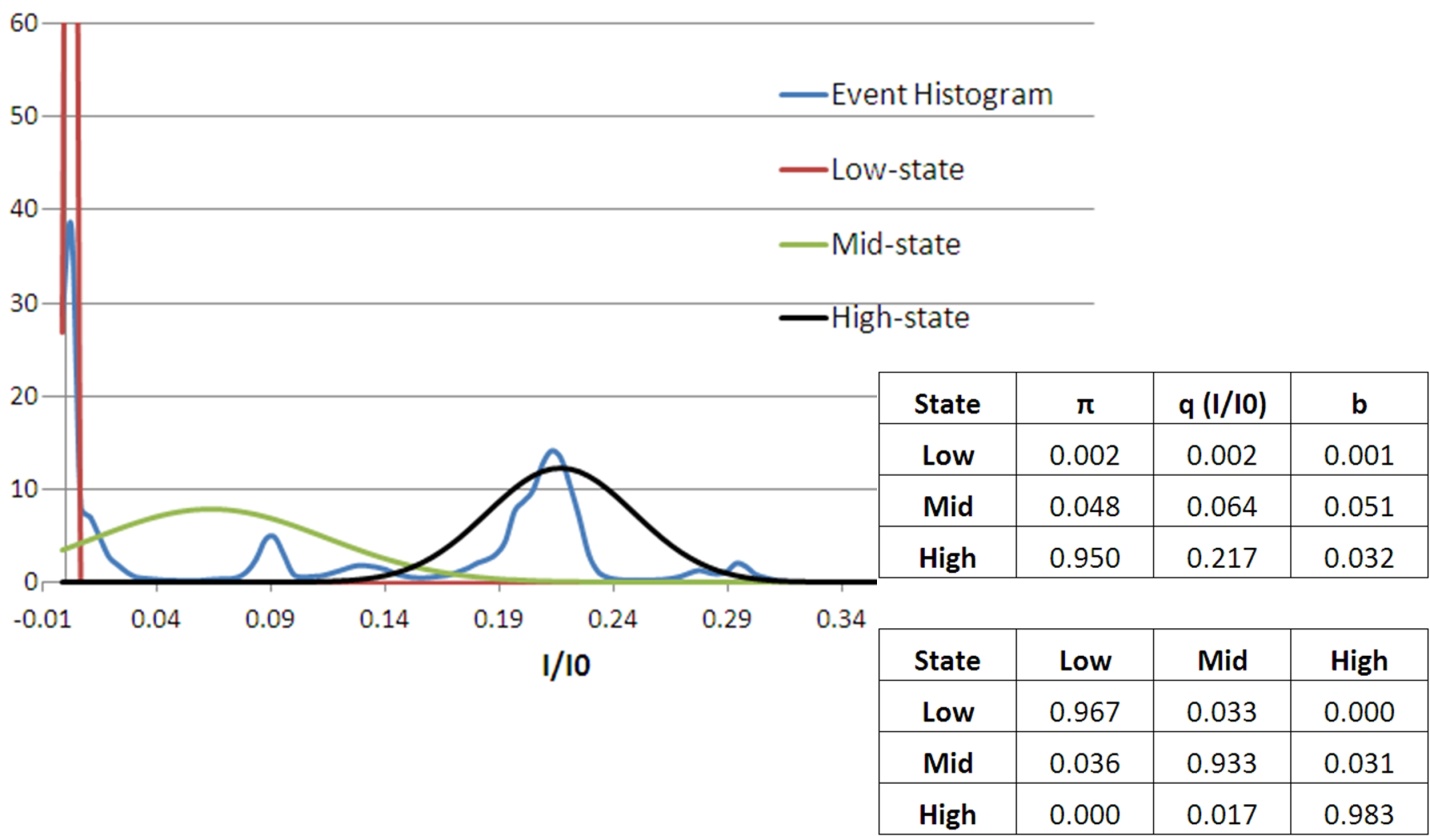


**Figure S3 – KKRR-ShPrP (120-232) event histogram and optimal HMM model (case 2). (Top left)** KKRR-ShPrP(120-232) event histogram of the training data (blue), with the high (black Gaussian), mid (green Gaussian), and low states (red Gaussian), as defined by the optimal HMM model to the training data, overlayed. **(Bottom right)** The optimal three-state HMM model to the training set. Initial condition (*π*), I/I0 state levels (*q*), noise properties of each state (*b* – i.e. the standard deviation on the Gaussian for each state), and the state-to-state transition probability matrix (*A*).

**KKRR-ShPrP(120-232)-D178N**


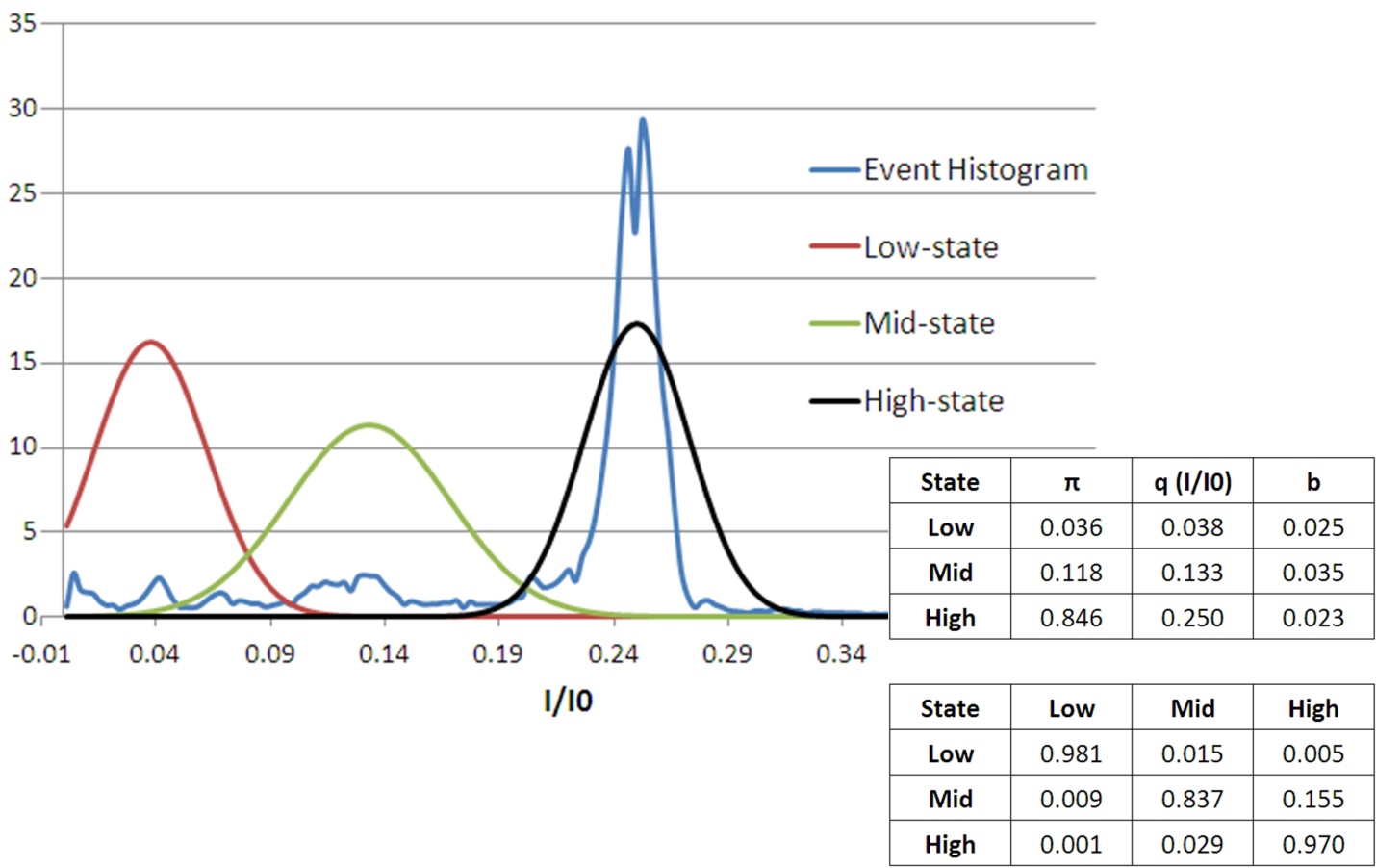


**Figure S4 – KKRR-ShPrP(120-232)-D178N event histogram and optimal HMM model (case 2). (Top left)** KKRR-ShPrP(120-232)-D178N event histogram of the training data (blue), with the high (black Gaussian), mid (green Gaussian), and low-states (red Gaussian), as defined by the optimal HMM model to the training data, overlayed. **(Bottom right)** The optimal three-state HMM model to the training set. Initial condition (*π*), I/I0 state levels (*q*), noise properties of each state (*b* – i.e. the standard deviation on the Gaussian for each state), and the state-to-state transition probability matrix (*A*).

**State-to-state transition rates**

Given the Viterbi path for each event the transition rates between states are determined by first constructing the log-state time distribution for each state over all events. The exit rate for a particular state (i.e. the rate to transition out of a given state) is given by:

$k_{exit}=\left(1-\frac{N}{N_{tot}}\right)\left(\int e^{\ln\left(t\right)}p\left[\ln\left(t\right)\right]d\left[\ln\left(t\right)\right]\right)^{-1}$, (S2)

where *p[ln(t)]* is the log-state time distribution for the given state (i.e. the normalized histogram of log-state times). *N* is related to the fact that the last occupied state in those events that do not end in escape from the pore is not timed but is nevertheless used in the calculation of the exit rate for that particular state. *N* is the number of instances, for a particular state, that were not timed and *N_tot_* is the total number of instances of that state. Given the exit rate for a particular state the state-to-state transition rate is then given by,

$$k_{i\to j}=p_{j}k_{exit}^{i}=\frac{n_{j}}{n_{tot}}k_{exit}^{i}$$

where $k_{i\to j}$ is the transition rate from state *i* to *j*, $k_{exit}^{i}$ is the exit rate out of state *i*, and *p_j_* is the probability of transitioning into state *j* from state *i*. *p_j_* is given by the number of transitions into state *j* from state *i* (*n_j_*) divided by the total number of transitions out of state *i* (*n_tot_*), which includes escapes from the pore from state *i*.

For the mid-state transition rates given in figure 6 (bottom left) and figure 6 (bottom right) the mid-state event times are first separated into two groups:

Group 1: Mid-state event times whereby the mid-state was entered from the high-state

Group 2: Mid-state event times whereby the mid-state was entered from the low-state.

Exit rates for each group and state-to-state transition rates are then determined as given above.
